# Management of Hand Avulsion Injury: A Case Report of a 39-Year-Old With Partial Soft Tissue Avulsion

**DOI:** 10.7759/cureus.30882

**Published:** 2022-10-30

**Authors:** Gana Josiah, Azer Thomas, David A Onilede, Eseleigbemen S Imafidon, Boma E Jacks, Ukaejiofo Echezona, Onyekwere Agbaeze, Okelue E Okobi

**Affiliations:** 1 Plastic and Reconstructive Surgery, Federal Medical Center Keffi, Keffi, NGA; 2 Surgery, University of Maiduguri Teaching Hospital, Maiduguri, NGA; 3 Plastic Surgery, Federal Medical Center Keffi, Keffi, NGA; 4 Family Medicine, Arizona State University, Tempe, USA; 5 Family Medicine, Lakeside Medical Center, Belle Glade, USA

**Keywords:** partial avulsion injury, hand function, skin reattachment, interest in hand and microsurgery, skin avulsion, hand injury

## Abstract

Hand injuries occur distal to the wrist crease in the upper limb. Infrequently life-threatening, but with the potential for severe disabilities and far-reaching effects. Hand injuries are common among productive adults. The initial stage in managing hand injuries is identifying the affected structures, followed by determining the urgent procedures required to restore full function. For example, suppose the mechanism of injury, the composition of the avulsed tissue, the extent of damage to the underlying tissues, and the dimension, pattern, and orientation of the avulsed tissue are favorable. In that case, successful reattachment of the avulsed tissue can be achieved. In this article, we describe a 39-year-old woman who underwent immediate repair of a partial avulsion of the radial aspect of her right hand with an almost satisfactory outcome.

## Introduction

The hand is a very important part of the body - functionally, structurally, and psychologically. Next to the brain, the hand is the greatest asset to man. Hands play an important role in maintaining body image and sense of identity as well as serving as an organ of communication, among other things [1]. Hand injuries occur in the region of the upper limb distal to the wrist crease [1-5]. They are rarely life-threatening but carry the potential for serious handicaps with far-reaching consequences, as hand injuries are more common in the productive age group [2-3]. Epidemiology of the hand injury may vary from one community to another depending on the occupation and industrial activities in that location. Preventive methods for hand injuries depend on the epidemiology of the injuries in the particular environment [4]. About 5% to 10% of patients seen in the emergency department of hospitals are due to hand injury. Such injuries result from a number of causes and can occur at home, on the road, in offices, in workshops, or even in the field of play or entertainment [2-3]. The main objective when treating hand injuries is to retain as much function as possible through well-managed primary care [1-3]. In our environment, most patients pay for their treatment. As a result, treatment may suffer delay or be completely abandoned, where funds are not immediately available to the patients [2-4]. In this case report, we followed the management of a lady who suffered a right-hand injury from a road traffic accident to identify the outcome of early reattachment of the avulsed tissue and the cost of care.

## Case presentation

**Figure 1:**
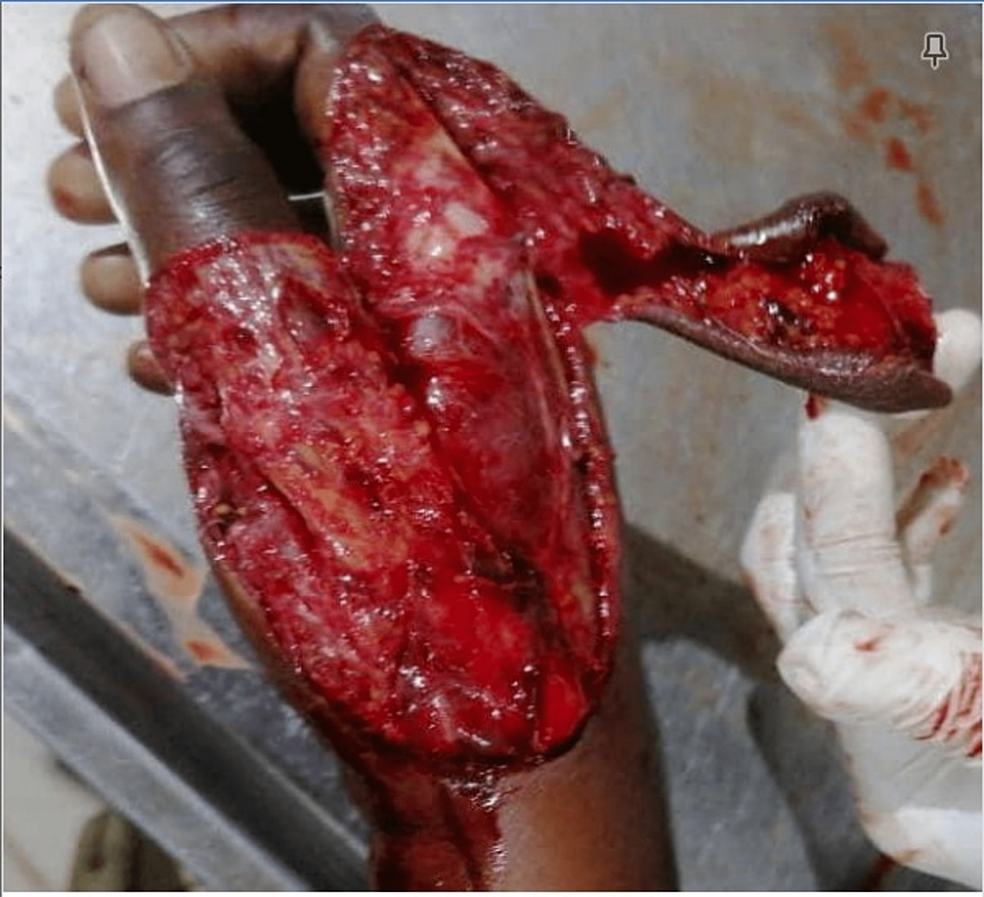
Immediate post-trauma right-hand avulsion injury

The patient is a 39-year-old female trader who was brought to the accident and emergency one hour following a motorcycle accident. She was an unprotected passenger, along with three of her children, on a motorcycle that was hit by another motorcycle and fell underneath a heavy-duty truck, with the loss of life of one of her children seated on the front of the motorcycle. There was no loss of consciousness or bleeding from any craniofacial orifices. However, she suffered an injury to the right hand. The patient was conscious and alert, oriented in time, place, and person. Examination of the right hand revealed a partial soft tissue, irregularly shaped dorsal avulsion on the radial, involving the thumb and index finger, measuring approximately 14cm x 10cm (see Figure 1 below). The partially avulsed tissue was distally based, hanging by a thin base on the radial aspect of the index finger. The hand felt warm to the touch, with slight swelling. When compared to the opposite hand, no muscle atrophy was seen. Cascade sign was negative, there was no crepitus, and the range of motion, though limited by pain throughout the fingers and wrist for flexion, extension, and radio-ulnar deviation, was intact (metacarpophalangeal joint (MCP): 0° extension to 80° of flexion, proximal interphalangeal (PIP) joint: 0° extension to about 100° of flexion, distal interphalangeal joint (DIP): 0° extension to about 55° of flexion). All dermatomes retained two-point differentiation. The radial nerve, medial nerve, and ulnar nerve were all intact. Vascular pulses were intact for a radial pulse and ulnar pulse, with a good capillary refill. Because of the patient's pain threshold, Allen's test was not done. There were no signs of fracture of the hand bones. Examination of other systems was normal.

**Figure 2:**
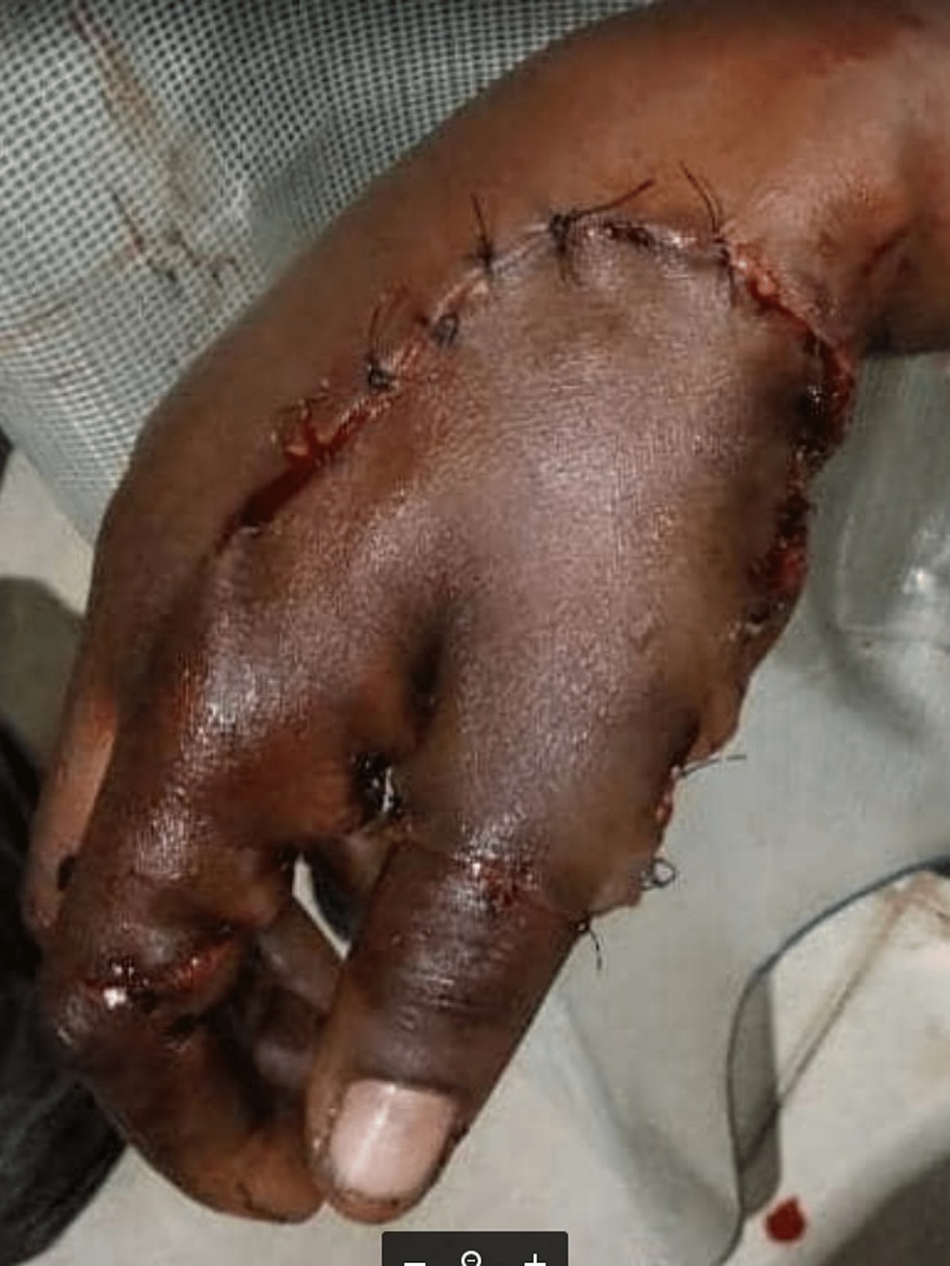
Immediate post-trauma right-hand avulsion injury repair

X-ray of the hand did not reveal any fracture of the hand bones. Packed cell volume was 38% at presentation. She was given 500ml intravenous normal saline fluid and had hemostasis secured with a pressure dressing. Tetanus prophylaxis, analgesia, and empirical antibiotics commenced according to local protocol. After examination of the avulsed tissue, which contained the skin and subcutaneous layer, with some part of the fascia over the hand muscles, consent was obtained to close off the wound with the distally based avulsed flap by reattachment (see Figure 2 below).

**Figure 3:**
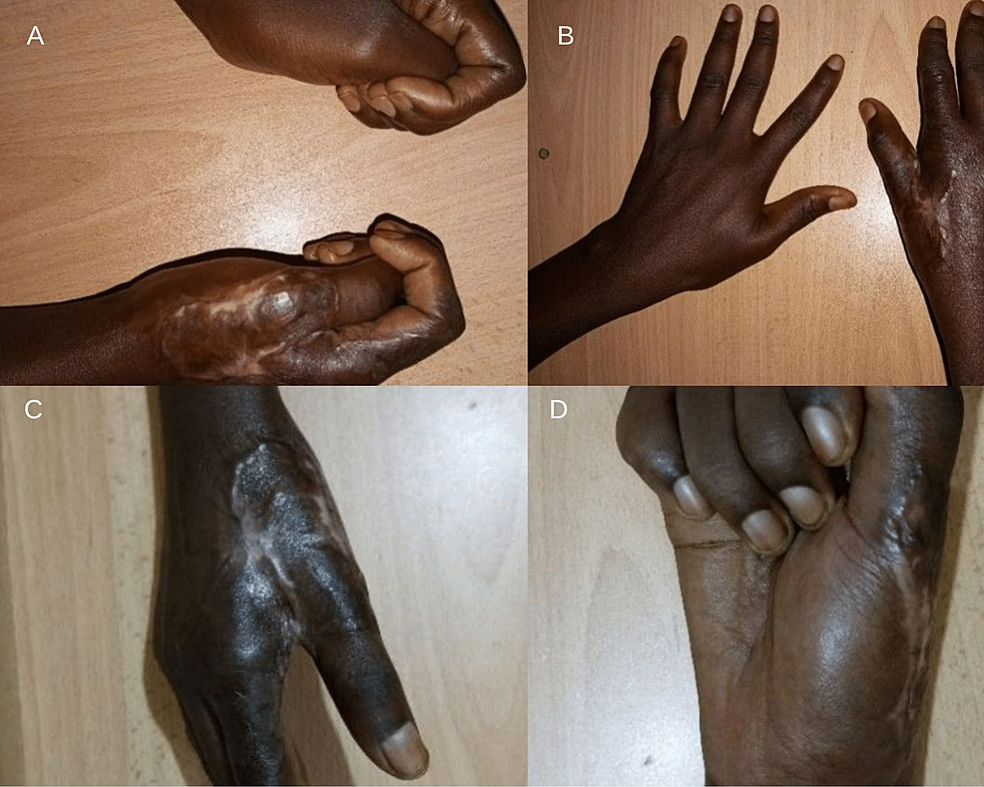
Avulsion injury repair eight weeks follow-up findings A. Posterior later view (with normal hand); B. Posterior view; C. Posterior lateral view; D. Anterior view

The wound was irrigated with one liter of normal saline, and the avulsed tissue was defatted to the dermis and sutured with prolene 3/0 to cover the hand defect using wide awake local anesthesia with no tourniquet (WALANT). A pressure dressing was applied over the reattached avulsed tissue, and the dressing was monitored daily for strikethrough stain. Satisfactory flap survival was observed after close monitoring. The hand was kept elevated on a collar and cuff. She was maintained on analgesics, broad-spectrum antibiotics, multivitamins, and the local wound dressing protocol followed every alternate day. She commenced physiotherapy on the 5th day following wound review. She was discharged home on the 7th day post-op. The reattached avulsed tissue/skin survived largely with minimal areas of skin loss just at the base of the thumb measuring approximately 3cm x 2cm, that healed after regular dressing. There was also resultant mild stiffness of the metacarpophalangeal joints of the thumb and index finger, which was corrected with physiotherapy. Healing was complete after eight weeks with the return of hand function (see Figure 3 below). A contracture was observed in the first web space, which the patient admitted did not significantly affect her quality of life or use of the right hand as her dominant hand.

## Discussion

Generally, hand surgeons all over the world agree that the goal of treatment of hand injury is to restore an aesthetically pleasing, painless, tactile, mobile, stable finger that can sense pain, temperature, pressure, steroidogenesis, and fine touch [1-4,6-7]. In managing hand injuries, following identifying the injured structures, the examiner must decide what immediate steps should be taken toward restoring full function. Reattachment of the avulsed tissue back in place can be achieved with desirable outcomes if the mechanism of injury, composition of the avulsed tissue, the extent of damage to the underlying tissues, and dimensions of the avulsed tissue are assessed and feasible [3,5].

Cheung et al. [7] highlighted the general principles in the approach to traumatic hand injuries with the first step as to perform a thorough history and physical examination, assessing and documenting important items such as the mechanism and time of injury, hand dominance, tetanus status, and the patient’s occupation and baseline function. During the physical examination, a thorough inspection with comparison to the uninjured hand should be performed, observing abnormal positioning, angulation or rotational deformity, or scissoring should be noted. The motor function should be documented, and independent tests of tendons and ligaments should be performed. Assessment of neurovascular status should include testing capillary refill and moving 2-point discrimination, depending on the nature of the injury. Anterior-posterior, lateral, and oblique X-ray scans might be required to rule out fractures, dislocations, and foreign bodies. Managing traumatic hand injuries involves reducing and immobilizing fractures, obtaining post-reduction X-ray scans, obtaining soft tissue coverage, preventing and treating infection, and ensuring tetanus prophylaxis [7].

Extremity degloving injuries usually result from run-over injuries or industrial accidents. The skin is avulsed from the deeper tissues due to the shearing force exerted over the skin following the entrapment of body parts in roller machines and conveyor belts in case of industrial accidents. Similar injury results following the crushing of the limb between the moving wheel and the road in case of run-over injuries. Though such injuries can occur in any part of the body, extremities are more commonly involved. Avulsion amputations are a severe form of these injuries where usually the projecting body parts get accidentally trapped in rotating machine shafts, and the tissues, in turn, are avulsed and detached to a variable extent [5].

Otene et al. observed that road traffic crashes were the second commonest cause of hand injuries [1]. The reasons for the high incidence of road crashes were given as poor road maintenance cultures as well as the use of poorly maintained vehicles and motorbikes as the main cause of road traffic crash in Nigeria, while other reasons may include reckless driving and poor knowledge of road signs. Usually, the very active age groups are commonly affected by these injuries, disabling a lot of the workforce as injuries may affect the patient’s dominant hand, thereby affecting full return to work [1]. This is similarly observed in our patient, who could not return to her normal daily activities throughout the first four weeks post-injury.

Successful replantation of avulsed digits, scalp, penile skin and even composite facial tissues has been reported. Skin graft harvesting from the avulsed flap has also been described; even composite flaps can be harvested from avulsed and amputated tissues that are not suitable for replantation but will need microsurgical transfer for their reattachment [3, 5, 6-10]. However, these techniques will require a longer admission duration and overall care cost.

In western countries, the majority of hand lacerations can be treated on an outpatient basis. The primary purpose of wound and laceration management is to avoid infection, detect if a nerve injury has occurred, manage tendon lacerations, and achieve a cosmetically acceptable result with the highest degrees of function and patient satisfaction [8-10]. Our patient had the benefit of early presentation; after assessment of the avulsion injury, it was determined that reattachment of the avulsed tissue and modification to improve its survival would be the most beneficial for the patient’s early return to full function. This was to be done under close monitoring to observe wound healing progress and prevent infection; as a result, the patient had to be admitted. The outcome was satisfactory with a short hospital stay, the need for more extensive surgery, and the overall cost of care.

## Conclusions

Avulsion injury of the hand is common following road traffic accidents. The tissue avulsed should be assessed to determine if replantation is possible and will result in better or similar outcomes as with more invasive interventions that may be available to preserve aesthetics and function. The avulsed tissue was modified and replanted in the presented case to cover the wound. This helped in reducing the patient’s hospital stay to seven days, morbidity, and enabled early return of function.
